# Anti-PABPC1 Co-Immunoprecipitation for Examining the miRNAs Directly Targeting the 3′-UTR of *EED* mRNA

**DOI:** 10.1371/journal.pone.0103695

**Published:** 2014-08-01

**Authors:** Yi Hu, Kun-Lun Yin, Xu Ma, Hong-Fei Xia

**Affiliations:** 1 Reproductive and Genetic Center, National Research Institute for Family Planning, Beijing, China; 2 Graduate School, Peking Union Medical College, Beijing, China; 3 Chinese Academy of Sciences Key Laboratory of Pathogenic Microbiology and Immunology, Institute of Microbiology, Chinese Academy of Sciences, Beijing, China; University of Toronto, Canada

## Abstract

MicroRNAs (miRNAs) are small, noncoding RNA molecules that regulate post-transcriptional gene expression by base pairing with partially complementary sequences within target messenger RNAs (mRNAs). Although the target genes and the precise biological functions of individual miRNAs remain largely unknown, miRNAs have been implicated in diverse biological processes, including both normal and pathological states. As a single stranded mRNA can be directly targeted by multiple miRNAs, and as the target sites may exist in the 3′-untranslated region (UTR), 5′-UTR, or the coding regions, it is essential to develop an effective method to identify the full-scale miRNA regulatory pattern of each particular gene. In this study, we employed a biochemical approach to identify the miRNA profiles that regulate the expression of embryonic ectoderm development (EED) protein by using anti-PABPC1 ribonucleoprotein (*RNP*) co-immunoprecipitation (Co-IP). The full length *EED* mRNA was subcloned into an expression vector and transiently transfected into a Flag-PABPC1 stable expression cell line. Subsequent to cross-linking and an anti-Flag *Co-IP*, the miRNAs that directly targeted *EED* were identified. We found that the best time point to distinguish the positive miRNAs from the background was 18 hours after the plasmid transfection. As expected, the miRNAs that directly target *EED* were found to interact with *EED* mRNA through the miRNA-induced silencing complex (miRISC). Meanwhile, the *EED* mRNA was bound by Flag-PABPC1. This method depends on the integrity of the miRISC complex and achieves greater efficiency when ultraviolet irradiation is used for the process of cross-linking. By using anti-PABPC1 RIP, we identified *EED* to be a new target gene of miR-16; a finding further confirmed using a dual-luciferase assay. In summary, our data indicate that anti-PABPC1 RIP is a validated and direct biochemical method to provide data about specific miRNA-mRNA interactions, as well as global miRNA patterns regulating the mRNAs.

## Introduction

MicroRNAs (miRNAs) are key regulators of gene expression that repress messenger RNA (mRNA) translation at the post-transcriptional level [1]. To exert their regulatory functions, miRNAs assemble into miRNA-induced silencing complexes (miRISCs), minimally comprising an argonaute protein (AGO) and a protein of the GW182 family [2], [3]. It is now widely accepted that a primary determinant for miRNA binding usually involves perfect, consecutive Watson-Crick base pairing between the target mRNA 3′-untranslated region (UTR) and the miRNA at position 2–7 in the 5′ end of the mature miRNA [4]. Recently, more and more reports have indicated that the sequences interacting with the miRNA seed region also exist in the coding regions and 5′-UTRs of the mRNAs [5], [6]. As the expression of one gene can be directly repressed by hundreds of various different miRNAs, developing a method to identify the miRNAs that target a specific mRNA sequence would be incredibly useful in unveiling the full-scale regulated effect of biologically important genes at the post-translational level.

The miRNAs regulate gene expression through translational repression and/or mRNA deadenylation and decay [7], [8]. Although the molecular mechanisms involved in the coordination of these different steps of the pathway remain elusive, it is possible that the formation of multi-protein complexes will play an important role in the dynamics of the miRNA-mediated regulation. In addition to the argonaute proteins (AGOs), the GW182 proteins also play key roles in miRNA-mediated repression [9]–[11]. Humans carry three GW182 paralogs (known as TNRC6A, B, and C, respectively), whereas *Drosophila melanogaster* (Dm GW182) carries only one family member of this protein. GW182 proteins function as scaffold proteins for the assembly of silencing complexes on mRNA targets. Accordingly, they interact with AGOs through an N-terminal argonaute-binding domain. There are reports indicating that a direct interaction of GW182 with AGOs is critical for the miRNA-mediated translational repression and mRNA decay [12]. Mammalian GW182 proteins interact with poly (A)-binding protein C1 (PABPC1) and the CCR4–NOT/PAN2-PAN3 deadenylase complex through a C-terminal silencing domain to promote deadenylation [1], [13]–[17]. Although the formation of this complex is not essential for the miRNA-mediated translational repression [18], PABPC1 acts as a crucial miRNA coactivator in the miRNA-induced mRNA decay process.

PABPC1 is a multifunctional protein with a variety of roles in mRNA translation and stability. In humans, the PABPs comprise a small nuclear isoform and a conserved gene family that displays at least 4 functional proteins: PABPC1, inducible PABP (iPABP or PABPC4); ePABP (embryonic PABP); and PABP3. Available data suggest that PABP1 and PABP4 are widely expressed, whereas expression of the other family members appears to be more tissue-restricted [19]. Besides the miRNA-mediated mRNA decay, PAPBC1 also plays a key role in the nonsense-mediated mRNA decay process [20].

Recently, anti-AGO immunoprecipitation has been used to study the global pattern of mRNAs that are recruited to miRISCs in response to particular miRNAs [21], [22]. Although defects exist, anti-AGO ribonucleoprotein (*RNP*) immunoprecipitation (RIP) has been developed as one of the most powerful methods to study the targetome of miRNAs. Since PABPC1 is another important molecule that acts as a bridge between mRNAs and miRNAs, we sought to determine whether it could be used as a target protein to isolate miRNAs that target to a specific mRNA.

In this study, we systematically evaluated the anti-PABPC1 RIP method and found that the best time to perform the RIP assay was 18 h after the plasmid transfection. We also compared the results from the non-cross-linking, ultraviolet (UV) cross-linking, and formaldehyde-cross-linking groups, and found that an additional UV-cross-linking step could achieve higher efficiency and specificity. Using this method, we identified that the embryonic ectoderm development protein (EED) is a new target of miR-16. Although the amount of RNA extracted from the pellet is not enough to be detected in a microarray, this method can be improved and is expected to become a powerful tool for identifying miRNAs that target one gene or a group of genes.

## Results

### Feasibility study for the anti-Flag-PABPC1 RIP strategy

**Figure 1:**
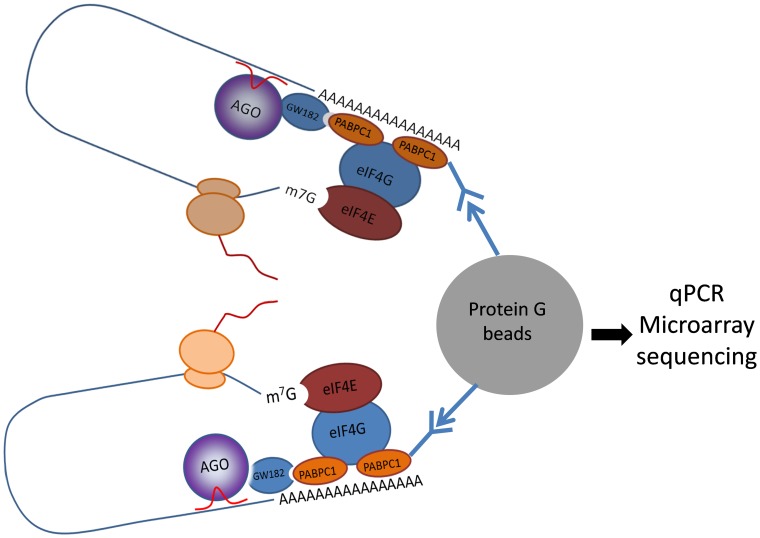
Schematic presentation of RNP immunoprecipitation. PABPC1 participates in the process of post-transcriptional regulation of gene expression by miRNA. Therefore, we used anti-PABPC1 to identify the miRNAs that target *EED* mRNA.

As shown in the schematic diagram in Fig. 1, our strategy was as follows. Full-length *EED* mRNA was overexpressed by transient transfection of the *EED* expression vector in a Flag-PABPC1 stable cell line. PABPC1 binds to almost all of the mRNAs that contain a polyA tail (in mammalian cells). Furthermore, miRNAs that directly target *EED* are expected to interact with *EED* through the miRISC component. Meanwhile, the *EED* mRNA should also be bound by Flag-PABPC1 at an appropriate time point. Cross-linking followed by anti-Flag *co-immunoprecipitation (Co-IP*) enables easy identification of the miRNAs that target *EED*.

**Figure 2:**
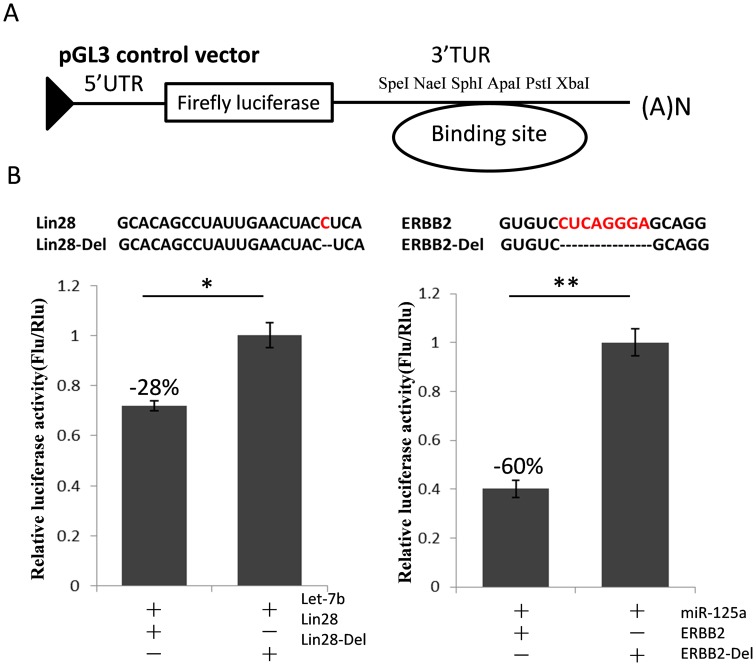
Construction of a quality control system. (A) A schematic diagram for the subcloning of the 3′-UTR of *Lin28* and *ERBB2* into the pGL3 vector. (B) HEK293T cells were co-transfected with Let-7b or miR-125a and the 3′-UTR of *Lin28* or *ERBB2* for the dual-luciferase assay. PRL-TK plasmid expressing the *Renilla* luciferase was used as a transfection control. The luciferase activity was detected 48 h after transfection, and the results were analyzed using the Student’s t-test. *P<0.05, **P<0.01.

To test whether our strategy was feasible or not, we first constructed a quality control system for RIP according to the protocol reported by Easow et al. [21]. Reporter plasmids were designed to carry the Let-7b miRNA recognition element of *Lin28* and the miR-125a recognition element of *Erbb2* (named as LIN28 and ERBB2, respectively) or their deleted sequence (LIN28-Del and ERBB2-Del, respectively) in the 3′-UTR (Fig. 2A). As expected, the Let-7b and miR-125a mimics inhibited the expression of the appropriate Fluc reporters (Fluc-LIN28 and Fluc-ERBB2, respectively), as compared with the control reporters (Fluc-LIN28-Del and Fluc-Erbb2-Del, respectively). These results showed that the quality control system for RIP was effective and usable.

**Figure 3:**
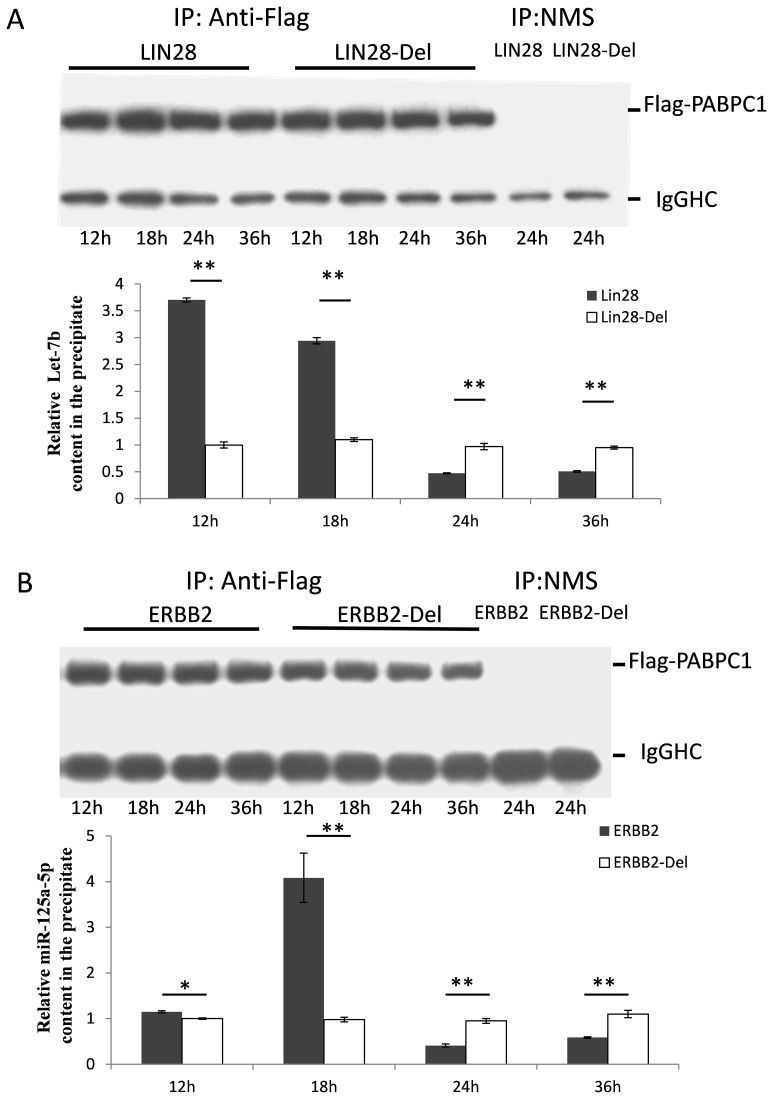
Time course and efficiency of the anti-Flag PABPC1 RIP. Plasmids containing the 3′-UTR of the wild type or mutant *Lin28* (A) or *ERBB2* (B) were transfected into the Flag-PABPC1 stable cell line. Anti-Flag PABPC1 RIP was used at four time points (12 h, 18 h, 24 h, and 36 h) after the transfection, and the total RNA in the cell lysate and precipitate was extracted using the TRIzol reagent. The miRNAs were detected by RT-qPCR and the results were analyzed using the Student’s t-test. *P<*0.05 was considered statistically significant. *P<0.05, **P<0.01.

We then used the quality control system to determine the optimal time point at which RIP is able to distinguish between the positive miRNAs and the background. First, a FLAG tag was introduced into the C-terminus of the PABPC1 protein and a stable HEK293T cell line expressing FLAG-PABPC1 was generated by puromycin selection. The immunopurification of the PABPC1-containing protein complexes was performed with an anti-FLAG antibody. The co-IP assay was optimized and validated by examining the specific pull down of Let-7b and miR-125a in the Flag-PABPC1 stable upregulated HEK293T cell line cotransfected with LIN28/LIN28-Del or ERBB2/ERBB2-Del, respectively. The samples were collected at four different time points (12 h, 18 h, 24 h, and 36 h) after the last step of transfection. A western blot was used to detect the amount of FLAG-PABPC1 in the precipitate. As shown in Fig. 3, FLAG-PABPC1 was specifically isolated with the anti-FLAG antibody, but not with the non-immunized mouse serum. Mature Let-7b and miR-125a were detected by real-time quantitative polymerase chain reaction (RT-qPCR), using the Taqman miRNA detection reagents. Although the expression level of the FLAG-PABPC1 proteins was the same in all the lysates, Let-7b (Fig. 3A) or miR-125a (Fig. 3B) expression was highly enriched in the anti-FLAG co-IP from the cell lysates transfected with the wild type *Lin28* or *Erbb2* mRNA 12 h and 18 h after transfection, when compared to the site deletion group. In particular, after 18 h of transfection, Let-7b and miR-125a were both enriched more than 3 times in the wild type mRNA transfection group than in the control group. Therefore, we chose this time point for the final experiment.

### Integrity of the miRISC complex is necessary for recruiting miRNAs

**Figure 4:**
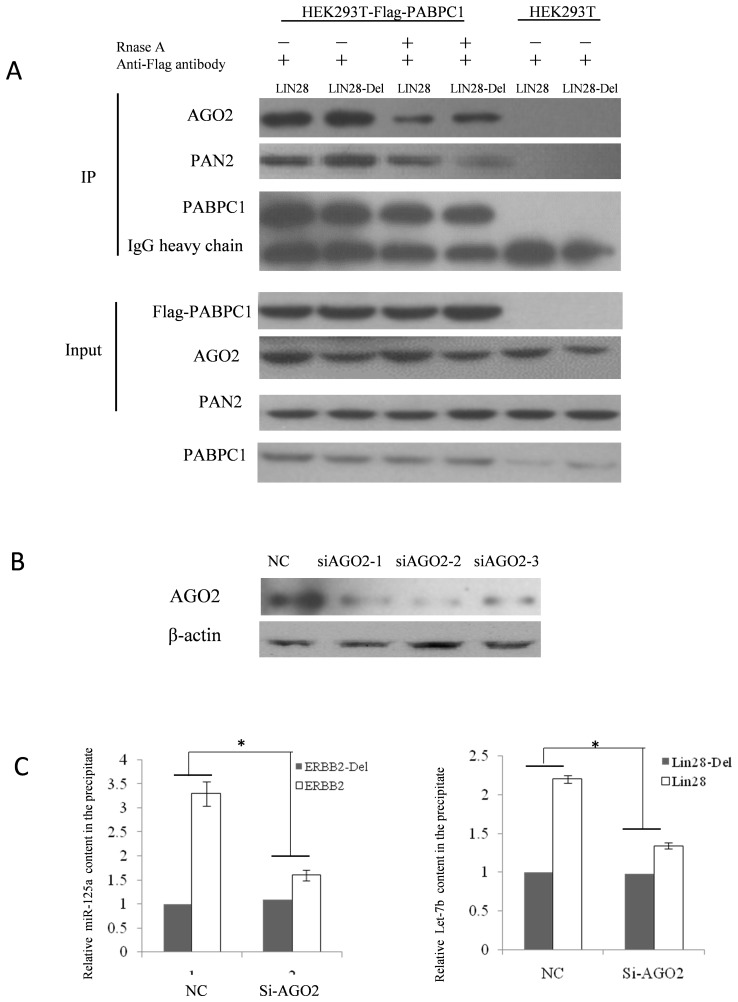
The integrated RISC complex is needed for miRNA recruitment. (A) Western blot analysis of the co-IPed products. Co-IPs were performed on cells transfected with the reporter plasmid LIN28 or LIN28-Del. As expected, the Flag-PABPC1 proteins were co-IPed with the anti-Flag antibody. Meanwhile, the anti-Flag antibody could not pull down the wild type PABPC1 in the HEK293T cell lysate. AGO2 and PAN2 were detected in the co-IPed products using anti-PAN2 and anti-AGO antibodies, respectively. RNase A was added to determine whether the co-IPed RISC-related components were affected by RNA degradation. In the RNase A-treated groups, the amounts of AGO2 and PAN2 were reduced, implying that the binding interactions between PABPC1 and AGO2 or PABPC1 and PAN2 are partially mRNA-dependent. (B) Knock down of endogenous AGO2 using siRNA. HEK293T cells were transfected with AGO2 siRNAs. At the end of the transfection (48 h), the cells were lysed and AGO2 expression was detected with a western blot. The knockdown effect was most effective in siAGO2-2. (C) Comparison of the miR-125a/Let-7b contents among the AGO2 knockdown groups and control groups. Cells were transfected with AGO2 siRNA. A scramble sequence and non-meaning short RNA was used as control. Cells were subsequently (after 48 h) divided into two dishes and transfected with the wild type or mutant LIN28 or ERBB2 plasmids, respectively. Anti-Flag co-IP was performed 18 h after the transfection and miR-125a and Let-7b were detected by RT-qPCR. The results were analyzed with the Student’s t-test and *P<*0.05 was considered statistically significant.

Independent miRNAs cannot inhibit gene expression. To exert their regulatory functions, they assemble into miRISC complexes. In other words, to confirm that the miRNAs recruited by FLAG-PABPC1 co-IP are functional, we needed to confirm that the endogenous miRISC complex could be indeed co-immunoprecipitated (co-IPed). We detected AGO2, the core unit of the miRISC complex, with a western blot. As shown in Fig. 4A, endogenous AGO2 was detected in the co-IP products. Interestingly, PAN2, the core component of the PAN2-PAN3 mRNA deadenylation complex, was also co-IPed by FLAG-PABPC1. However, in the RNase A treatment group, the amounts of co-IPed AGO2 and PAN2 were partially reduced, which means that the interactions between PABPC1, AGO2, and PAN2 are partially dependent on RNA molecules. In mammals, AGO1–4 constitute the AGO subfamily of AGO proteins, but only AGO2 participates in the two mRNA post-translational inhibition pathways (siRNA and miRNA). Therefore, we knocked down AGO2 expression using small interfering RNA (siRNA). As shown in Fig. 4B and C, the expression of AGO2 was reduced, and the amount of miR-125a and Let-7b recruited by the PABPC1 co-IP was significantly decreased as compared to the control group. The results indicated that the FLAG-PABPC1 co-IP method is integrated in a miRISC-dependent manner.

### Recruiting miRNAs that target EED by RIP

EED is an important member of the polycomb group family. EED, together with EZH2 and SUZ12, forms the polycomb repressive complex 2, which catalyzes the trimethylation of histone H3 lysine 27. There are two isoforms of the EED protein in humans that are similar in all aspects, except for the lengths of the respective C-terminal regions. The mRNA of the shorter EED isoform contains a longer 3′-UTR region (758 nt). As stated in our previous report, miR-30b and miR-30c can target the 3′-UTR of *EED* instead of miR-181b [23]. There are some differences in the prediction results obtained using TargetScan and Miranda, two popular web prediction tools. We used *EED* mRNA (the shorter isoform) to test the efficiency of our method. Meanwhile, as UV irradiation [17] and formaldehyde treatment [24] are the two most frequently used cross-linking methods to study protein-nucleic acids interactions, we decided to add UV cross-linking and formaldehyde cross-linking to develop a better method that showed higher specificity and efficiency.

**Figure 5:**
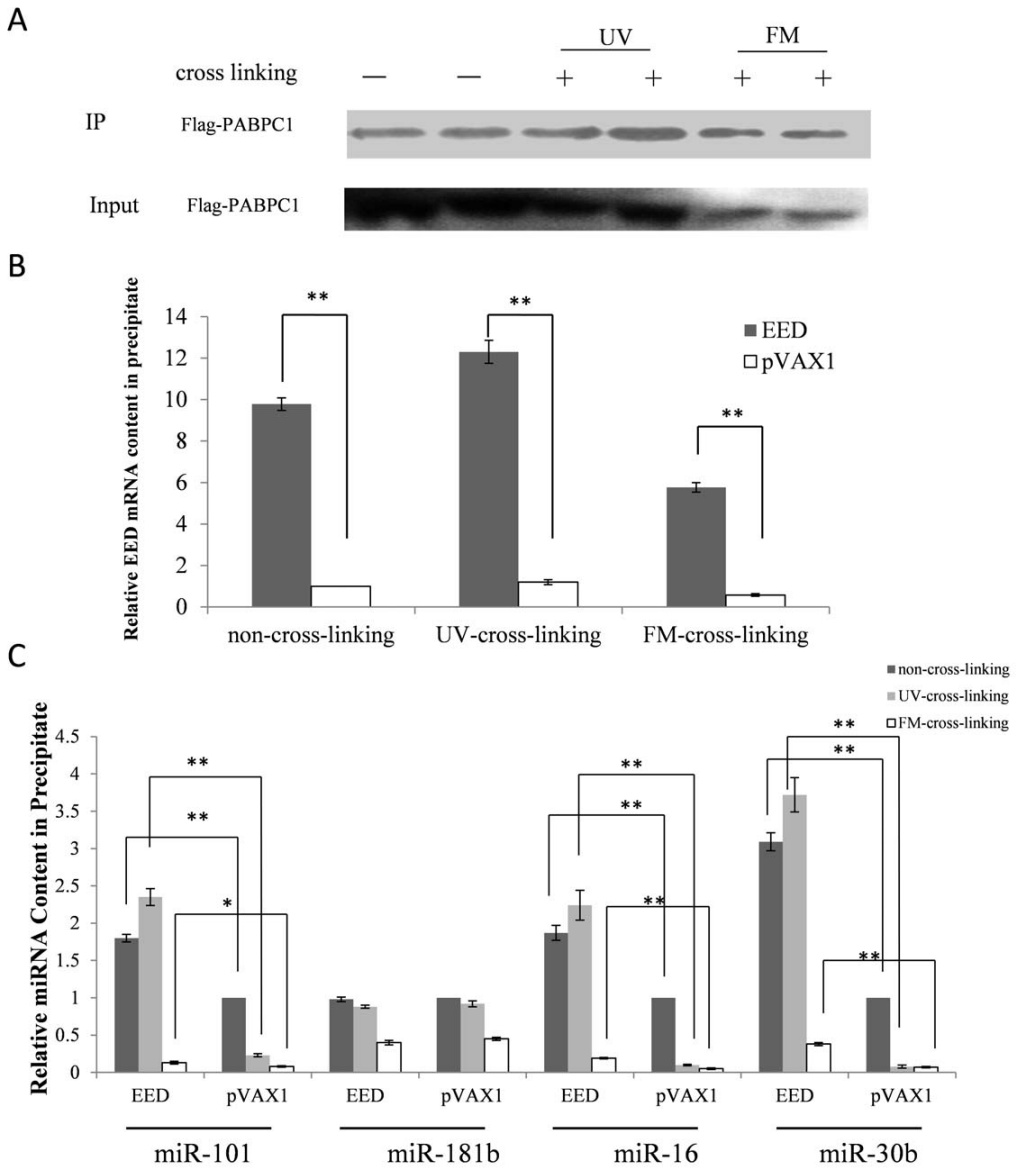
Anti-PABPC1 RNP immunoprecipitation for identifying miRNAs that target *EED*. (A) Western blot analysis of co-IPed products. Cells were transfected with the EED expression vector, with empty pVAX1 as the control. Cells were treated with 150 mJ/cm^2^ UV (lanes 3, 4) or formaldehyde (FM) (lanes 5, 6) 18 h after transfection to cross-link the protein-RNA complex. The cells were then lysed and co-IPed. As expected, the FLAG-PABPC1 proteins were immunoprecipitated with the anti-FLAG antibody and the FLAG-PABPC1 protein contents were nearly the same in the non-cross-linking and UV cross-linking groups. However, the FLAG-PABPC1 amount was reduced in the lysate and precipitate of the formaldehyde treatment group compared to those of the non-cross-linking and UV cross-linking groups. (B) The *EED* mRNA levels were detected by RT-qPCR and the results were analyzed using the Student’s t-test. *P<*0.05 was considered statistically significant. (C) The levels of four selected miRNAs in the precipitate were detected by RT-qPCR. Results were analyzed by the Student’s t-test. *P<0.05, **P<0.01.

The *EED* coding sequence along with the 3′-UTR was subcloned into the pVAX1 vector, which does not express the mRNA of the other resistance genes in eukaryotic cells. The plasmid pVAX1-EED was transfected into the FLAG-PABPC1 stable cell line, using null pVAX1 as a control. Western blot from the co-IPs showed that FLAG-tagged PABPC1 was specifically associated with the anti-FLAG antibody and the amount was nearly the same in the non-cross-linking and UV-cross-linking groups, but was slightly reduced in the formaldehyde cross-linking group (Fig. 5A). The result of real-time PCR showed that the *EED* mRNA was 9.9-, 12.3-, and 9.7-fold enriched in the pellet in the pVAX1-EED groups compared to that in the pVAX1 groups in the non-cross-linking, UV-cross-linking, and formaldehyde cross-linking groups, respectively. However, the *EED* mRNA yield in the formaldehyde cross-linking group was found to be reduced (Fig. 5B). Meanwhile, expression levels of the mature miR-101, miR-16, and miR-30b were nearly the same in the lysate of the pVAX1-EED group as compared to the pVAX1 group (Fig. S1), and were enriched in the precipitate of the pVAX1-EED groups (Fig. 5C). However, the levels of miR-101, miR-16, and miR-30b in the UV cross-linking group were higher than those in the non-cross-linking and formaldehyde cross-linking groups, respectively (Fig. 5C and S1, respectively). The result indicates that UV cross-linking is the best cross-linking method for the RIP experiment, and that miR-101 and miR-16 may target *EED* directly.

### The results of RIP were confirmed with the dual-luciferase reporter system

**Figure 6:**
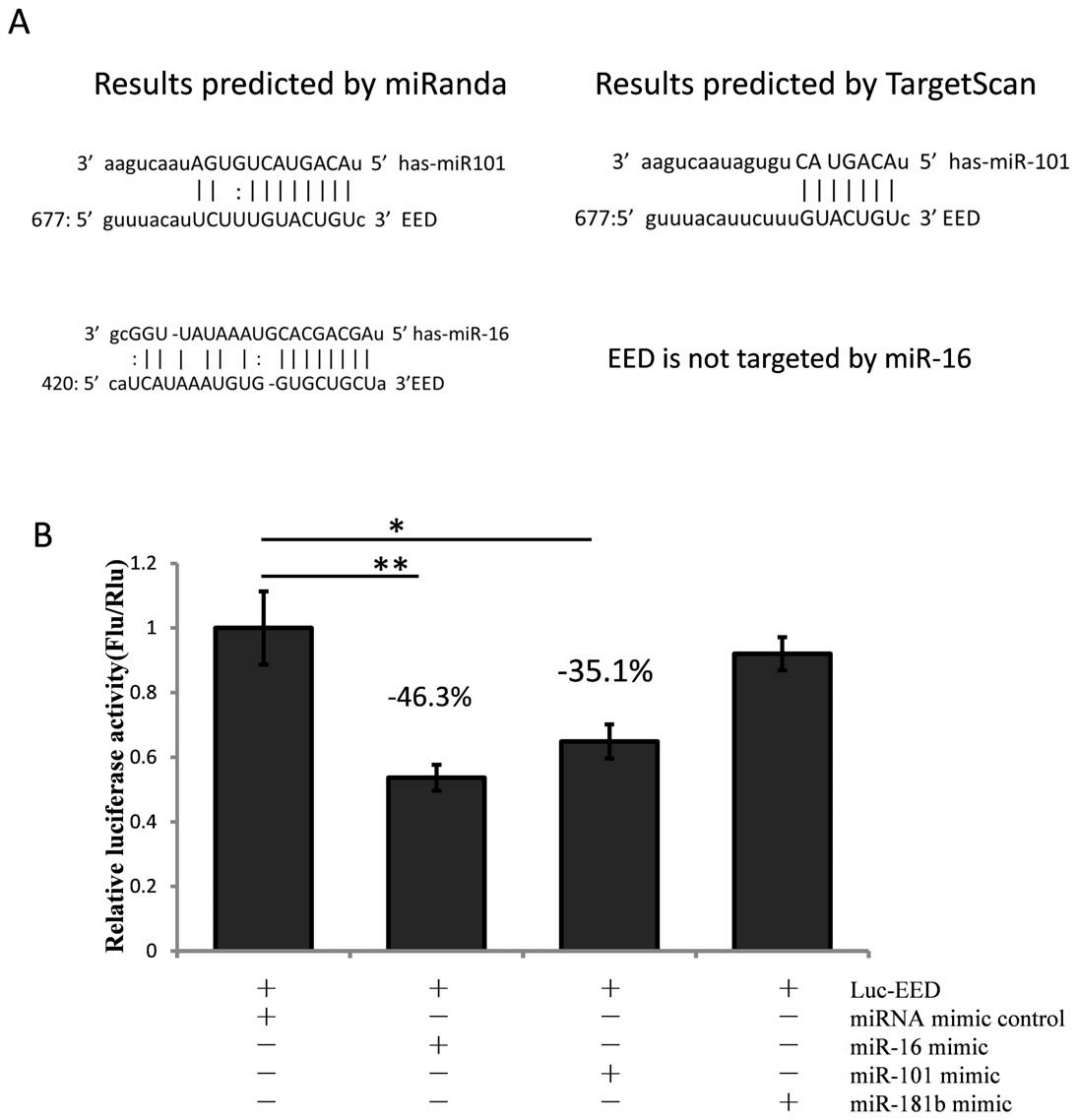
MiR-16 and miR-101 suppress EED expression through targeting to the 3′-UTR of *EED*. (A) The predicted results of miRanda indicated that *EED* is targeted by miR-16 and miR-101, whereas the TargetScan online tools indicated that there could be a direct interaction between miR-16 and *EED* mRNA. (B) Confirmation of the relationship between *EED* and miR-101/miR-16. Cells were co-transfected with the miRNA mimic control, the miR-101 mimic, the miR-16 mimic, or the miR-181b mimic, and the Luc-EED for the dual-luciferase assay. PRL-TK containing *Renilla* luciferase was co-transfected with the 3′-UTR of *EED* for data normalization. *P<0.05, **P<0.01.

**Figure 7:**
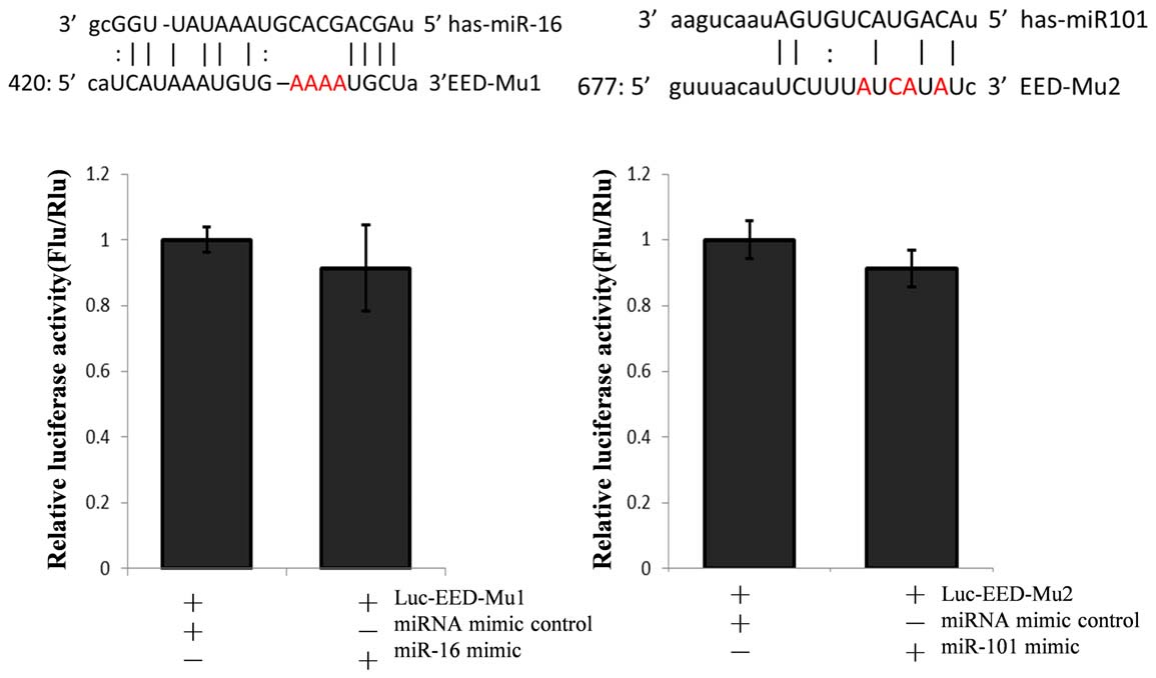
Identifying the target sites of miR-16 and miR-101 in the 3′-UTR of *EED*. The predicted binding site of miR-16 and miR-101 in the 3′-UTR of *EED* was mutated (designated as Luc-EED-Mu1 and Luc-EED-Mu1, respectively). HEK293T cells were transfected with miR-16 or 101, and the mutant *EED* 3′-UTR reporter vectors separately, with pRL-TK as the transfection control. The luciferase activity was detected 48 h after transfection. Results were analyzed with the Student’s t-test. *P<0.05, **P<0.01.

To verify the results reported above, we used a dual-luciferase reporter system to detect the repressive effect of miR-101 and miR-16 on *EED* expression. A human wild-type *EED* 3′-UTR fragment was subcloned downstream of the firefly luciferase reporter gene in the pGL3 control vector (designated as Luc-EED). As shown in Fig. 6, the relative firefly luciferase activity was reduced by about 46.3% or 35.1% when cells were co-transfected with Luc-EED and miR-16 or miR-101, respectively. When the seed sequence was mutated, the repression effects nearly disappeared (Fig. 7). This result indicates that miR-16 and miR-101 target the *EED* 3′-UTR directly. This result is also in complete agreement with the result obtained from RNP immunoprecipitation.

## Discussion

MicroRNAs represent an important class of short, non-coding RNA molecules that regulate gene expression post-transcriptionally. According to miRBase Release 21, the human genome encodes 2588 mature miRNAs, which may target at least 60% of the protein-coding genes [25], [26].

Although researchers have developed many different bioinformatics tools to predict the interactions between miRNAs and mRNAs, the identification of miRNA targets has nonetheless proven to be challenging. As experimental data are always at the heart of these prediction algorithms, bioinformatics tools need to be modified with the increasing accumulation of experimental data. Until recently, anti-AGO2 RIP was reported as the most powerful method to study the targetome of miRNAs. As one mRNA may interact with hundreds of miRNAs, developing an effective method to identify the miRNAs that target a specific gene is useful in promoting the process of unveiling the post-transcriptional regulation pattern of genes.

The poly (A)-binding proteins (PABPs), which are found to bind to the poly (A) tail of eukaryotic mRNA, are required for poly (A) shortening and translational initiation. Recently, PABPC1 was identified as a cofactor in the miRNA-mediated translational repression process [13]–[15]. Therefore, we chose PABPC1 as the target molecule to perform RIP. When one mRNA is expressed at high levels in the cells, it could recruit the miRNAs that target it as an miRNA sponge [27]. Meanwhile, the target mRNA molecules are bound by PABPC1 at an appropriate time point. Therefore, the anti-PABPC1 antibody could be effectively used to recruit the miRNAs.

In this study, we first chose four time points to determine the most efficient time point for this method. We found that Let-7b and miR-125a were both more than 3-times enriched in the wild type mRNA transfection groups 18 h after transfection. However, at the time points of 24 h and 36 h post-transfection, Let-7b and miR-125a expression was reduced in the wild type mRNA transfection groups. To explore why more miRNAs appeared in the precipitate in negative controls at the later time points, we detected the microRNAs and firefly luciferase mRNA contents at the four time points. As shown in Fig. S2, the expression of luciferase mRNAs showed an increasing trend 12 h after transfection, but the miRNA contents started decreasing gradually. There are reports claiming that the overexpression of target mRNAs can expedite the miRNA decay rate [28]. Based on our data and on those of previous reports, we hypothesize that a large part of miR-125a/Let-7b falls into the miRNA decay or the recycling pathway 24 h and 36 h after transfection because of high target mRNA expression. Therefore, we obtained fewer miR-125 and Let-7b transcripts in wild type mRNA than that in the mutation groups.

MiRNAs are posttranscriptional regulators of gene expression that associate with AGO and GW182 proteins to repress translation and/or promote mRNA degradation. GW182 was originally identified in human cells as the antigen recognized by the serum of a patient suffering from motor and sensory neuropathy [29]. GW182-containing foci became known as GW bodies (GWBs). Subsequent studies showed that GWBs coincide with the mRNA-processing bodies or P-bodies [30]. GW182 proteins provide a docking platform through which deadenylase complexes gain access to the poly (A) tail of the miRNA targets to promote their deadenylation. GW182 proteins recruit the PAN2-PAN3 and CCR4-CAF1-NOT deadenylase complexes through direct interactions with PAN3 and NOT1 [31]. GW182 proteins also directly bind to PABP and interfere with its function, thus leading to the silencing of the target mRNAs [32]. In this study, we co-IPed PAN2 and AGO2 using FLAG-PABPC1. The quantities of these two molecules were reduced in the RNase A-present conditions. FLAG-PABPC1 seems to have the same function as that of endogenous PAPBC1, and the indirect binding between PABPC1 and PAN2 or AGO2 is partially RNA dependent.

There is evidence that the protein-RNA complex can form post-lysing during immunoprecipitation analyses [33]. As shown in Fig. S3, miR-125a and Let-7b were recruited by FLAG-PAPBC1 from other plate cells. Therefore, choosing a proper cross-linking method to obtain a snapshot of the FLAG-PABPC1-mRNA-miRNA complex is necessary to obtain results that best reflect the actual mRNA and miRNA interaction conditions *in* *vivo*. In this study, we compared the UV cross-linking and formaldehyde cross-linking methods. Although these two methods were both successful at recruiting miRNAs that target *EED*, it was challenging to completely lyse the formaldehyde-treated cells. Therefore, the amounts of FLAG-PABPC1 and miRNAs obtained were reduced in the formaldehyde-treated groups.

As part of this study, we have developed a method to identify miRNAs that target to a specific mRNA. We chose four time points to perform the RIP assay and found that the best time to distinguish the miRNAs that interact with target mRNA from the background was 18 h after plasmid transfection. We have developed an incredibly useful method to unveil the post-transcriptional regulation pattern of one specific gene or a group of genes (and not simply the miRNAs). However, this method has its own limitations. For example, the amount of recruited low-abundance miRNAs cannot be detected using conventional high-throughput methods, and the mRNA overexpression may also cause some false-positive results. Furthermore, when the mRNA we wish to study is present at high concentration, the efficiency of this method is significantly reduced.

For further improvement, we suggest the use of aptamers to enhance the specificity and efficiency of our proposed method. Aptamers are usually oligonucleic acid or peptide molecules that bind to a specific target molecule. They could be artificial or natural. An aptamer recognizes a short sequence that can be added to the target mRNA to perform a pull-down experiment. Using this improvement, the target mRNA can be derived from the universal mRNA pool, and the specificity and efficiency of the new method can also be increased significantly.

In conclusion, we developed a method to identify miRNAs that target to a specific mRNA. Although limitations still exist, our method may be broadly used to study the post-transcriptional regulation patterns of target genes of miRNAs.

## Materials and Methods

### Molecular cloning

A partial double stranded nucleotide containing the 5′ endonuclease cleavage sites was obtained by annealing the primers 5′- TCGAGGGATCCGTCGACGGGCCCTGATCAGCGGCCGCG-3′ and 5′-AATTCGCGGCCGCTCATCAGGGCCCGTCGACGGATCCC-3′, respectively. The partial duplex was ligated into the XhoI/EcoRI sites of the pMSCV-puro vector (Clontech; Mountain View, CA, USA). The full length human *PABPC1* was amplified and subcloned into the BamHI/SalI sites of the modified pMSCV-puro vector, and the FLAG-tag coding sequence was added directly to the reverse primer.

To generate the 3′-UTR luciferase reporter, the partial sequence of the 3′-UTR from Lin28 and ERBB2 was subcloned downstream of the firefly luciferase gene in the pGL3-control vector (Promega; Madison, WI, USA).

To generate the EED expression vector, the *EED* coding sequence connected to the 3′-UTR was amplified and subcloned into the NheI/PmeI sites of pVAX1 (Invitrogen; Carlsbad, CA, USA).

### Stable cell line establishment

Stable cell lines expressing FLAG-PABPC1 were generated by transfection of HEK293T cells in six-well plates with 4 µg of the plasmid using Lipofectamine 2000 (Invitrogen; Carlsbad, CA, USA), according to the manufacturer’s protocol. Selection for the plasmid was applied using 0.5 mg/mL of puromycin (Amresco; Solon, OH, USA), beginning 48 h after the transfection. The puromycin-resistant cell population was tested for the expression of FLAG-PABPC1, by immunoblotting with the anti-FLAG antibody (Abmart; Shanghai, China).

### Immunoblotting

The samples for the immunoblots were separated by sodium dodecyl sulfate-polyacrylamide gel electrophoresis and transferred to a polyvinylidene fluoride membrane (Bio-Rad; Hercules, CA, USA), using the semi-dry technique. The primary antibodies used were anti-FLAG (Abmart; Shanghai, China), anti-AGO2 (Epitomics; Hangzhou, ZJ, China), anti-PAN2 (Proteintech; Wuhan, HB, China), and anti-β-actin (Santa Cruz Biotechnology Inc.; SantaCruz, CA USA). Peroxidase-conjugated secondary antibodies were also used, and the bound antibodies were visualized on an ECL System, according to the manufacturer’s protocol (Pierce; Appleton, WI, USA).

### Reporter assays

Two hundred nanograms of a firefly luciferase reporter plasmid, 50 ng of a *Renilla* luciferase expressing plasmid (Promega, Madison, WI, USA), and 500 ng of the miRNA-expression plasmid or the empty vector were transfected into HEK293T cells (24 wells). Transfections were performed in triplicate. A dual-luciferase assay system (Promega; Madison, WI, USA) was used 48 h post-transfection, according to the manufacturer’s instructions.

### Anti-PABPC1 co-IP

Anti-FLAG antibody-conjugated beads (Abmart; Shanghai, China) were rinsed three times with phosphate-buffered saline (PBS) and blocked with 0.5 mg/mL yeast RNA and 1 mg/mL bovine serum albumin (Amresco; Solon, OH, USA) for 30 min or more. Beads were then washed thrice in PBS, followed by two washes in the lysis buffer (25 mM Tris-HCl, pH 8.0, containing 150 mM NaCl, 2 mM MgCl_2_, 0.5% NP-40, and 5 mM dithiothreitol, respectively). Forty-eight hours after the final transfection, the cells were first rinsed twice in PBS, and were then lysed on ice for 10 min in a fresh lysis buffer containing protease inhibitors (1 tablet/10 mL lysis buffer; Complete Protease Inhibitor Cocktail Tablets, EDTA-free; Roche Applied Science) and RNasin (Promega; Madison, WI, USA). The cell lysates were then centrifuged at 10,000×*g* for 10 min at 4°C, and the supernatants were collected. The lysates were subjected to preclearance by incubation with pre-blocked Protein G beads at 4°C for 60 min. An aliquot of the lysate after the preclearance, but before any co-IP, was removed for determining the total RNA and protein levels. The remaining lysates were used for the co-IP with the anti-FLAG antibody at 4°C for 90 min. After the co-IP, the beads were washed as follows: twice with lysis buffer; thrice with the lysis buffer containing 900 mM NaCl and 1% NP-40; and twice more with lysis buffer. The beads were then transferred to a fresh tube and subjected to a final wash with the lysis buffer containing 0.05% NP-40. Following the washes, the beads were subjected to DNase treatment by incubating them with 250 mL of DNA digestion solution containing 40 mM Tris-HCl at pH 8.0, 10 mM MgSO_4_, 1 mM CaCl_2_, 200 U/mL RNasin, and 0.04 U/mL DNase I (Promega; Madison, WI, USA). The DNase I treatment was carried out at 37°C for 20 min with gentle shaking. An aliquot of beads was removed from each sample and mixed with 2X Laemmli sample buffer for western blot analysis. RNAs that co-IPed with the anti-FLAG antibodies were extracted using TRIzol reagent (Invitrogen; Carlsbad, CA, USA). The total RNA from the cell lysates was isolated using the same procedure, and was subjected to DNA digestion as described above.

For UV cross-linking treatment, the cells were irradiated with 150 mJ/cm^2^ of UV radiation before the RIP experiment, as described by Konig et al [34]. For the formaldehyde cross-linking treatment, formaldehyde was added directly to the medium (within about 10 min) to achieve a final concentration of 1%. The formaldehyde-treated cells were lysed by using lysis buffer containing 1% sodium dodecyl sulfate.

### Real-time PCR

A TaqMan miRNA assay was used to detect the contents of miRNAs. The single-stranded cDNA was synthesized using the TaqMan miRNA reverse transcription kit (Applied Biosystems; Foster City, CA, USA) and then amplified by using the TaqMan universal PCR master mix (Applied Biosystems; Foster City, CA, USA) together with the following miRNA-specific TaqMan MGB probes: Let-7b, miR-125a, miR-181b, miR-16, miR-30b, and miR-101 (Applied Biosystems; Foster City, CA, USA).

### Statistical analysis

The data were analyzed using the SPSS Statistical Package version 16. For the luciferase reporter assays and for the qPCR assays that had more than two groups, we used multiple comparison/post-hoc analysis of variance, and the results of two independent groups were compared using the Student’s t-test. P<0.05 was considered statistically significant.

## Supporting Information

Figure S1 The detection of mature miR-101, miR-16, and miR-30b in the cell lysate. Forty-eight hours after the transfection, the cells were first rinsed twice in PBS, and were either left untreated or subjected to cross-linking with UV or formaldehyde. Cells were lysed on ice for 10 min. An aliquot of the lysate after the preclearance, but before a co-IP, was removed for total RNA analysis. The expression of three selected miRNAs was detected by RT-qPCR. The results were analyzed with one-way ANOVA and *P*<0.05 was considered statistically significant.
(TIF)

Figure S2 Detection of miRNA expression in the cells transfected with the target mRNAs. The expression levels of the luciferase mRNAs were significantly elevated 12 h after the transfection. Meanwhile, the miRNA contents were gradually reduced.
(TIF)

Figure S3 Assessing the likely formation of a protein-RNA complex after cell lysis. (A) HEK293T cells were transfected with the wild type ERBB2/Lin28 expression vector or with the mutant vector. The cells were lysed 18 h after transfection. The cell lysate was mixed with the Flag-PABPC1 expression stable cell lysate and then subjected to co-IP. The total RNA was extracted and the miRNAs were detected using RT-qPCR. (B) HEK293T cells were transfected with the wild type ERBB2/Lin28 expression vector or with the mutant vector. The cells were then treated with 150 mJ/cm^2^ of UV radiation 18 h after transfection, and subsequently lysed. The cell lysate was mixed with the Flag-PABPC1-expressing stable cell lysate and then subjected to co-IP. The total RNA was extracted and the miRNAs were detected using RT-qPCR. The results were analyzed using the Student’s t-test. P<0.05 was considered statistically significant. *P<0.05, **P<0.01.
(TIF)
