# Supplementary figures and images for: Anti-PABPC1 Co-Immunoprecipitation for Examining the miRNAs Directly Targeting the 3′-UTR of EED mRNA
Source: PLoS One. 2014 Aug 1;9(8):e103695. doi: 10.1371/journal.pone.0103695 (PMC4118908; doi:10.1371/journal.pone.0103695)

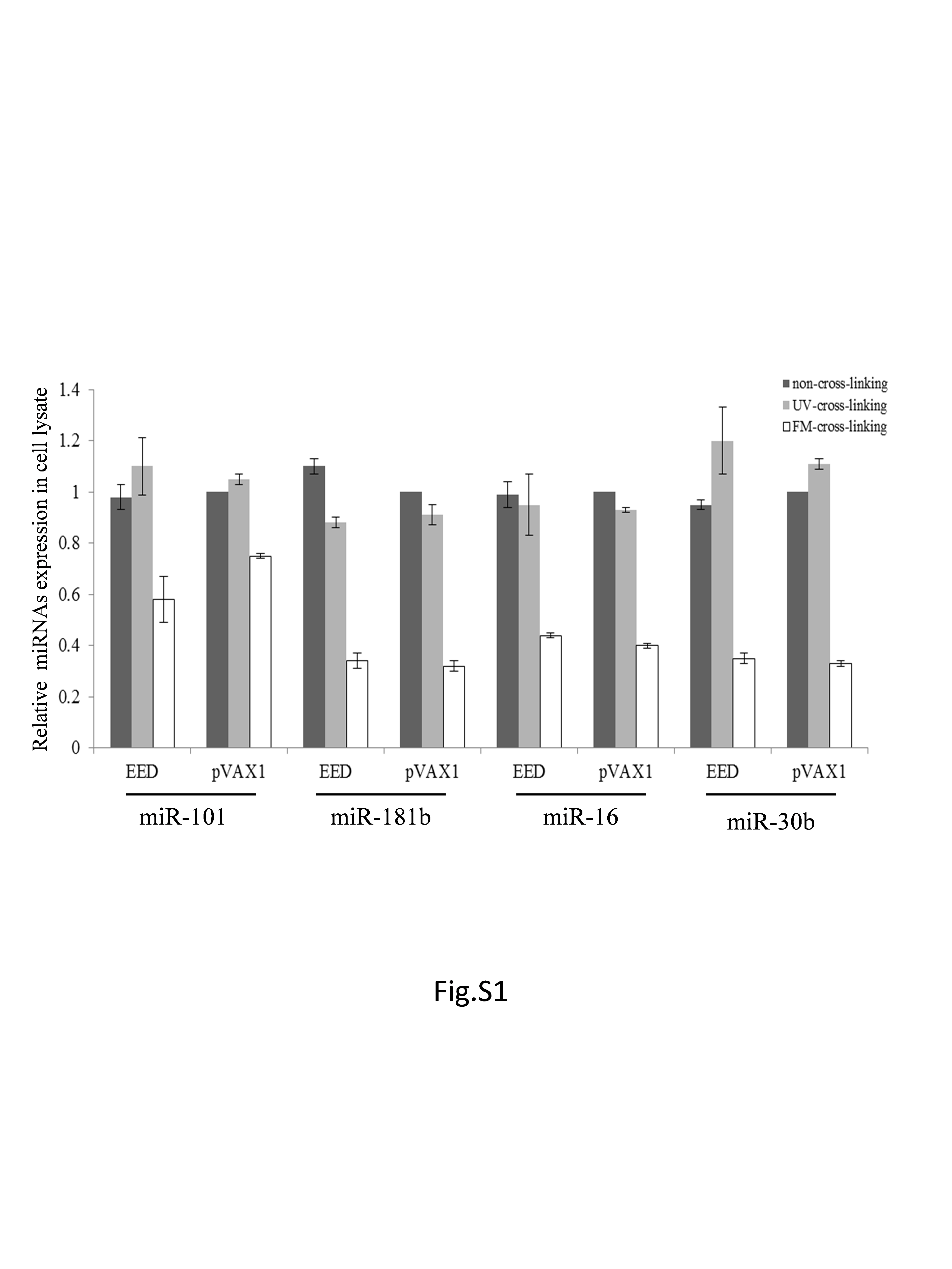

Supplement: Figure S1 — The detection of mature miR-101, miR-16, and miR-30b in the cell lysate. Forty-eight hours after the transfection, the cells were first rinsed twice in PBS, and were either left untreated or subjected to cross-linking with UV or formaldehyde. Cells were lysed on ice for 10 min. An aliquot of the lysate after the preclearance, but before a co-IP, was removed for total RNA analysis. The expression of three selected miRNAs was detected by RT-qPCR. The results were analyzed with one-way ANOVA and P<0.05 was considered statistically significant. (TIF) [file pone.0103695.s001.tif]

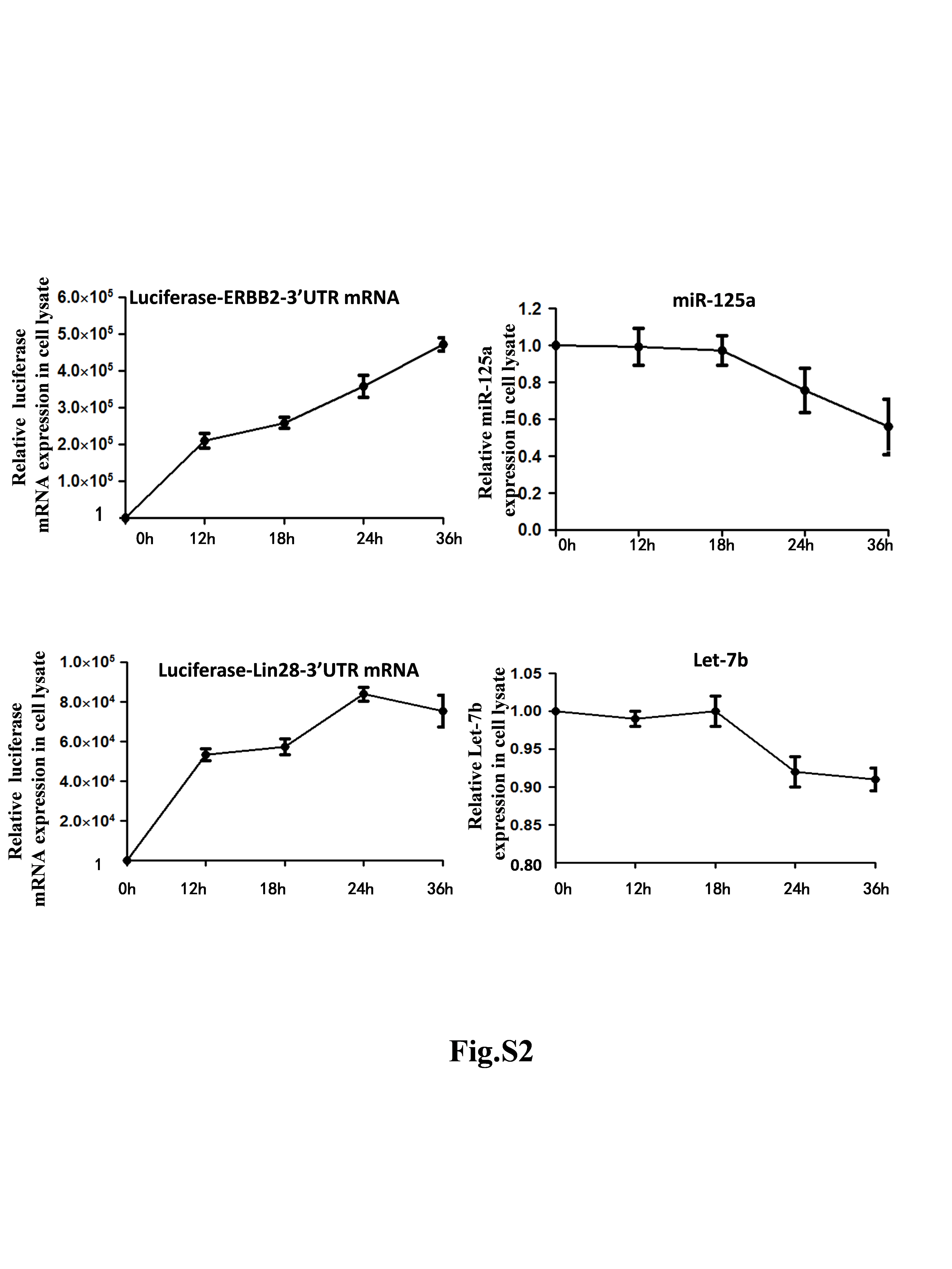

Supplement: Figure S2 — Detection of miRNA expression in the cells transfected with the target mRNAs. The expression levels of the luciferase mRNAs were significantly elevated 12 h after the transfection. Meanwhile, the miRNA contents were gradually reduced. (TIF) [file pone.0103695.s002.tif]

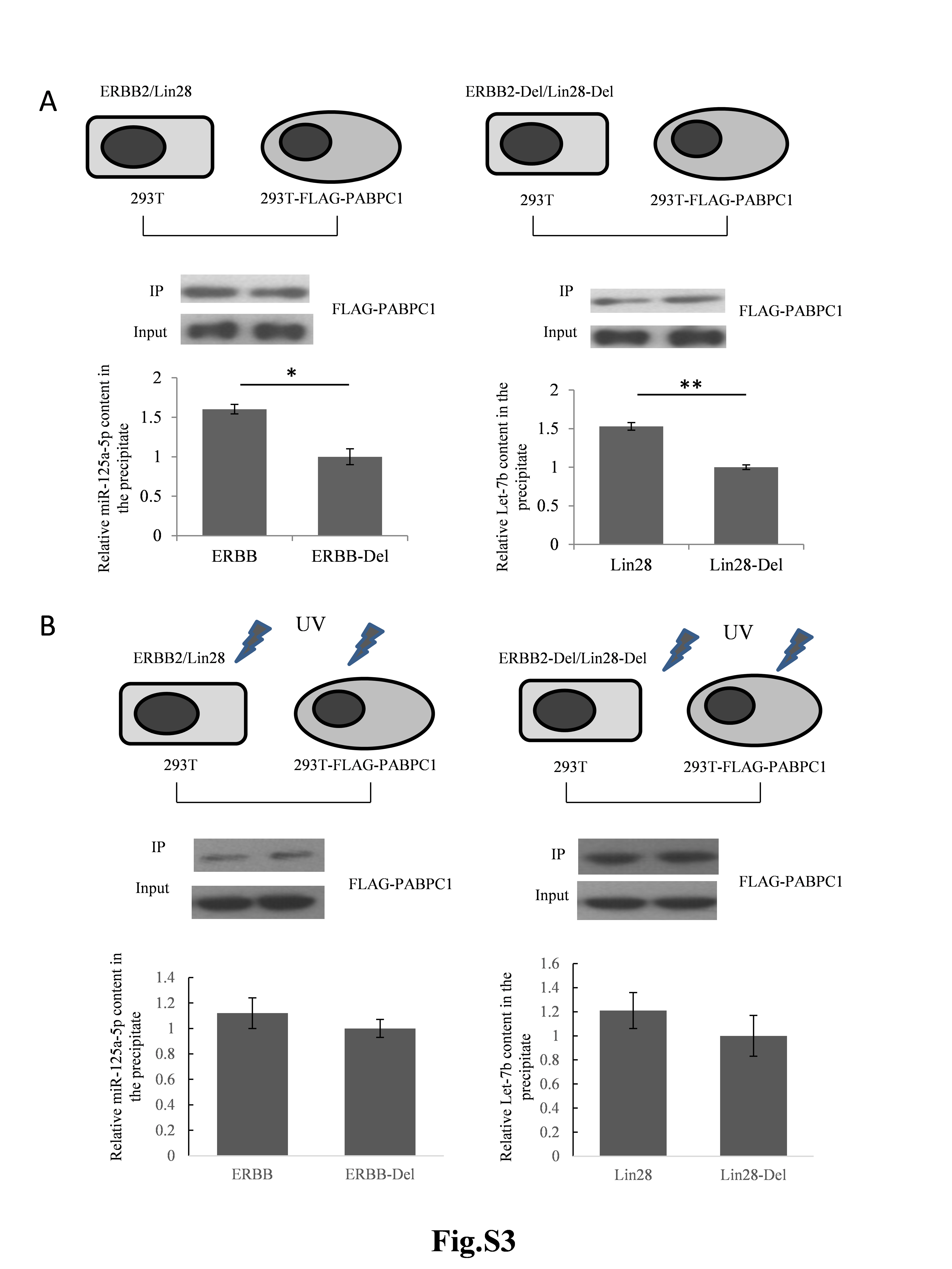

Supplement: Figure S3 — Assessing the likely formation of a protein-RNA complex after cell lysis. (A) HEK293T cells were transfected with the wild type ERBB2/Lin28 expression vector or with the mutant vector. The cells were lysed 18 h after transfection. The cell lysate was mixed with the Flag-PABPC1 expression stable cell lysate and then subjected to co-IP. The total RNA was extracted and the miRNAs were detected using RT-qPCR. (B) HEK293T cells were transfected with the wild type ERBB2/Lin28 expression vector or with the mutant vector. The cells were then treated with 150 mJ/cm2 of UV radiation 18 h after transfection, and subsequently lysed. The cell lysate was mixed with the Flag-PABPC1-expressing stable cell lysate and then subjected to co-IP. The total RNA was extracted and the miRNAs were detected using RT-qPCR. The results were analyzed using the Student’s t-test. P<0.05 was considered statistically significant. *P<0.05, **P<0.01. (TIF) [file pone.0103695.s003.tif]
